# Perinatal risk factors for neonatal encephalopathy: an unmatched case-control study

**DOI:** 10.1136/archdischild-2017-312744

**Published:** 2017-08-05

**Authors:** Cally J Tann, Margaret Nakakeeto, Barbara A Willey, Margaret Sewegaba, Emily L Webb, Ibby Oke, Emmanuel Derek Mutuuza, Donald Peebles, Margaret Musoke, Kathryn A Harris, Neil J Sebire, Nigel Klein, Jennifer J Kurinczuk, Alison M Elliott, Nicola J Robertson

**Affiliations:** 1 Clinical Research Department, London School of Hygiene & Tropical Medicine, London, UK; 2 MRC/UVRI Uganda Research Unit on AIDS, Entebbe, Uganda; 3 Institute for Women’s Health, University College London, London, UK; 4 Neonatal Medicine, Mulago University Hospital, Kampala, Uganda; 5 Uganda Women’s Health Initiative, Kampala, Uganda; 6 MRC Tropical Epidemiology Group, London School of Hygiene & Tropical Medicine, London, UK; 7 Department of Microbiology, Virology and Infection Prevention and Control, Great Ormond Street Hospital For Children NHS Trust, London, UK; 8 Institute for Child Health, University College London, London, UK; 9 National Perinatal Epidemiology Unit, Nuffield Department of Population Health, University of Oxford, Oxford, UK

**Keywords:** Neonatal Encephalopathy, Risk Factors, Uganda, Infection

## Abstract

**Objective:**

Neonatal encephalopathy (NE) is the third leading cause of child mortality. Preclinical studies suggest infection and inflammation can sensitise or precondition the newborn brain to injury. This study examined perinatal risks factor for NE in Uganda.

**Design:**

Unmatched case–control study.

**Setting:**

Mulago National Referral Hospital, Kampala, Uganda.

**Methods:**

210 term infants with NE and 409 unaffected term infants as controls were recruited over 13 months. Data were collected on preconception, antepartum and intrapartum exposures. Blood culture, species-specific bacterial real-time PCR, C reactive protein and placental histology for chorioamnionitis and funisitis identified maternal and early newborn infection and inflammation. Multivariable logistic regression examined associations with NE.

**Results:**

Neonatal bacteraemia (adjusted OR (aOR) 8.67 (95% CI 1.51 to 49.74), n=315) and histological funisitis (aOR 11.80 (95% CI 2.19 to 63.45), n=162) but not chorioamnionitis (aOR 3.20 (95% CI 0.66 to 15.52), n=162) were independent risk factors for NE. Among encephalopathic infants, neonatal case fatality was not significantly higher when exposed to early neonatal bacteraemia (OR 1.65 (95% CI 0.62 to 4.39), n=208). Intrapartum antibiotic use did not improve neonatal survival (p=0.826). After regression analysis, other identified perinatal risk factors (n=619) included hypertension in pregnancy (aOR 3.77), male infant (aOR 2.51), non-cephalic presentation (aOR 5.74), lack of fetal monitoring (aOR 2.75), augmentation (aOR 2.23), obstructed labour (aOR 3.8) and an acute intrapartum event (aOR 8.74).

**Conclusions:**

Perinatal infection and inflammation are independent risk factors for NE in this low-resource setting, supporting a role in the aetiological pathway of term brain injury. Intrapartum antibiotic administration did not mitigate against adverse outcomes. The importance of intrapartum risk factors in this sub-Saharan African setting is highlighted.

**What is already known on this topic?**

- Perinatal brain injury is the third leading cause of child mortality globally, with some of the highest burden seen in low-resource African settings.
- Preclinical studies suggest infection and inflammation can sensitise or precondition the term newborn brain to injury.
- Understanding which perinatal risk factors are associated with neonatal encephalopathy is key to developing interventions to prevent newborn deaths and disability.

**What this study adds?**

- Perinatal infection and inflammation are independent risk factors for neonatal encephalopathy in this African population, supporting a role in the aetiological pathway of term brain injury.
- Intrapartum antibiotic use, however, was not associated with improved neonatal outcome.
- The importance of other intrapartum risk factors in this setting is highlighted.

## Introduction

Birth complications and perinatal infections are leading contributors to neonatal mortality globally.^1^ Each year, peripartum complications contribute to more than one million cases of neonatal encephalopathy (NE) and around half a million survivors with neurological impairment.^2^ In sub-Saharan Africa, where access to skilled birth attendants and emergency obstetric intervention is often limited, the contribution of peripartum hypoxic events to NE is likely very high.^3^ Across other settings, however, a number of peripartum risk factors for NE have been identified,^4–7^ supporting a complex multifactorial model of brain injury.

Increasing evidence suggests the critical importance of a sensitising effect of inflammation in the pathogenesis of NE.^8 9^ In neonatal rodent studies, exposure to bacterial endotoxin has been found to increase vulnerability of the developing brain to injury, with a pathway involving stimulation of toll-like receptors, inflammatory responses, chemotaxis and cell death.^10^ Other preclinical studies have shown a temporal relationship between bacterial endotoxin and brain injury, with both sensitising and preconditioning effects seen.^11 12^ In clinical studies, factors associated with perinatal infection such as maternal fever and prolonged rupture of membranes, are associated with NE^5–7 10 13^; however, this may be mediated by the direct effect of hyperthermia itself on the developing brain as opposed to any underlying cause.^13^ Few clinical studies have examined the role of specific perinatal infections and inflammation as independent risk factors for NE, although an important role is hypothesised.^14^

Understanding which perinatal risk factors are associated with NE is key in developing interventions to prevent newborn deaths and disability. Despite a high burden of NE in sub-Saharan Africa, the role of infectious comorbidity, such as neonatal bacteraemia and chorioamnionitis, and the contribution of other risk factors in the aetiology of NE have been poorly defined. We conducted an unmatched case–control study among hospital-born newborns in Uganda aiming to identify risk factors for NE in resource-limited settings.

## Methods and materials

### Setting

Uganda is a low-income country with a neonatal mortality rate of 23 per 1000 live births.^15^ Mulago National Referral Hospital, in the capital Kampala, receives high-risk pregnancies from the city and surrounding areas. In 2012, more than 33 000 deliveries occurred on the low-risk (21%) and high-risk (79%) labour wards. Fetal monitoring is by intermittent auscultation and women are not routinely examined at the start of second stage. Assisted deliveries (ventouse or forceps) are rarely performed. A fifth of deliveries are by caesarean section. Intravenous fluids, antibiotics and oxytocin are available. Neonatal resuscitation, performed by midwives, includes oxygen and bag-mask ventilation. Care on the 80-bed special care baby unit (SCBU) includes simple continuous positive airway pressure ventilation (not mechanical ventilation), intravenous fluids, antibiotics and antiseizure medication. Blood gas estimation facilities are not available.

### Study design and recruitment

**Figure 1:**
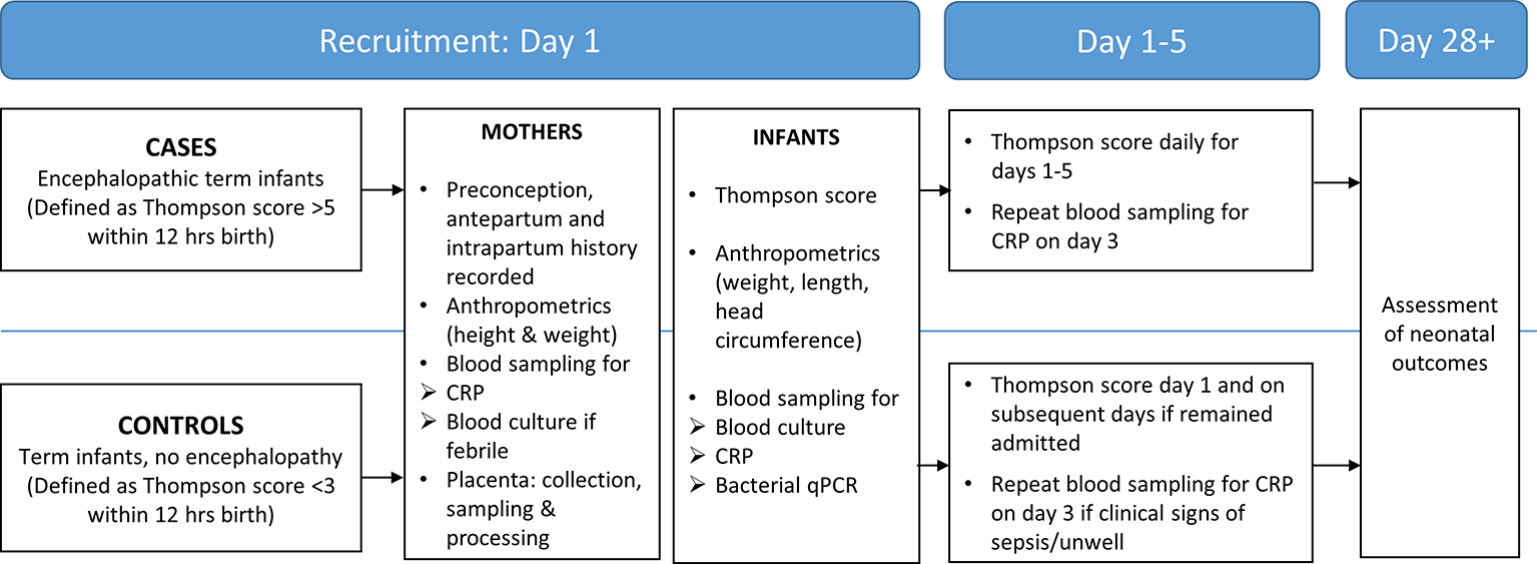
Diagram showing how infants moved through the study procedures. CRP, C reactive protein; qPCR, quantitative Polymerase Chain Reaction.

We conducted an unmatched case–control study between September 2011 and October 2012. Written informed parental consent was obtained. All term newborns admitted to the SCBU were examined for encephalopathy using the Thompson score.^16^ Cases were term newborns ≥37 weeks, with NE defined as a ‘Thompson score’ >5 within 12 hours of birth, as assessed by CJT or other study doctors. Neurological assessment was performed on recruitment for cases and controls and then daily for 5 days (cases only). Encephalopathy was graded (mild, moderate or severe) on the most severe day between days 1 and 5, per modified Sarnat classification,^17^ a scoring system used to grade the severity of hypoxic-ischaemic brain injury. Gestational age was assessed using last menstrual period or early obstetric ultrasound scan, and if unavailable based on external newborn examination.^18^ Infants were reviewed after discharge at 4–6 weeks of age to establish survival. Figure 1 describes how case and control infants moved through the study.

To reflect all hospital deliveries, controls were recruited in a ratio of 79:21 from the high-risk and low-risk wards, respectively. Control mothers and infants were systematically sampled from the labour ward admission book. Control infants were eligible for recruitment if term with Thompson score <3. Exclusion criteria (cases and controls) included prior antibiotics given to the infant (which would invalidate blood culture results), mother living >20 km from the hospital, out-born infants and no informed written consent. Infants with congenital abnormalities or other concomitant pathology were not excluded.

### Data collection

Information from antepartum, intrapartum and postpartum periods was collected using structured maternal interviews and from clinical records. Postnatal anthropometric measurements on mother (height, weight) and newborns (occipitofrontal circumference, birth weight (SECA 336 electronic scales, Hamburg, Germany)) were taken.

### Maternal and neonatal infection and inflammation

Maternal and neonatal blood was sampled at recruitment (<12 hours of delivery). Blood was stored at −80°C. Maternal and neonatal C reactive protein (CRP) was batch-tested (COBAS, Roche Diagnostics, Basel, Switzerland). Maternal HIV results were recorded from routine hospital testing.^19^

Blood culture and species-specific bacterial real-time PCR assays detected neonatal bacteraemia. Blood cultures (BACTEC) were performed for all case infants and for control infants with a clinical suspicion of sepsis. Isolated colonies were manually identified. Techniques for species-specific bacterial PCR and blood culture among the study cohorts have been published previously.^20^ Multiplexed PCR assays for the detection of bacteria considered pathogenic among newborns (group B Streptococcus, Pneumococcus, *Staphylococcus aureus*, group A streptococcus, Enterobacteriaceae sp) were performed on all cases and the first 101 control infants as a comparison group.^20^ CRP was measured as a marker of inflammation and presented according to centiles among control infants.

### Placental pathology

Placentas are not routinely collected and stored at Mulago Hospital, and we aimed to collect, process and report placental histology in a quarter of cases and controls. Placentas were collected when infant resuscitation was required (Apgar score <6 at 5 min) and for identified potential controls. Whole placentas were fixed (10% formalin), sampled according to standard protocols and processed (SurgPath, Kampala). Slides were reported by an experienced perinatal pathologist (NJS) at the Camelia Botnar Laboratories, Great Ormond Street Hospital, blinded to outcome and all clinical information. Histological chorioamnionitis and funisitis were defined according to standard criteria.^21^

### Statistical analysis

**Figure 2:**
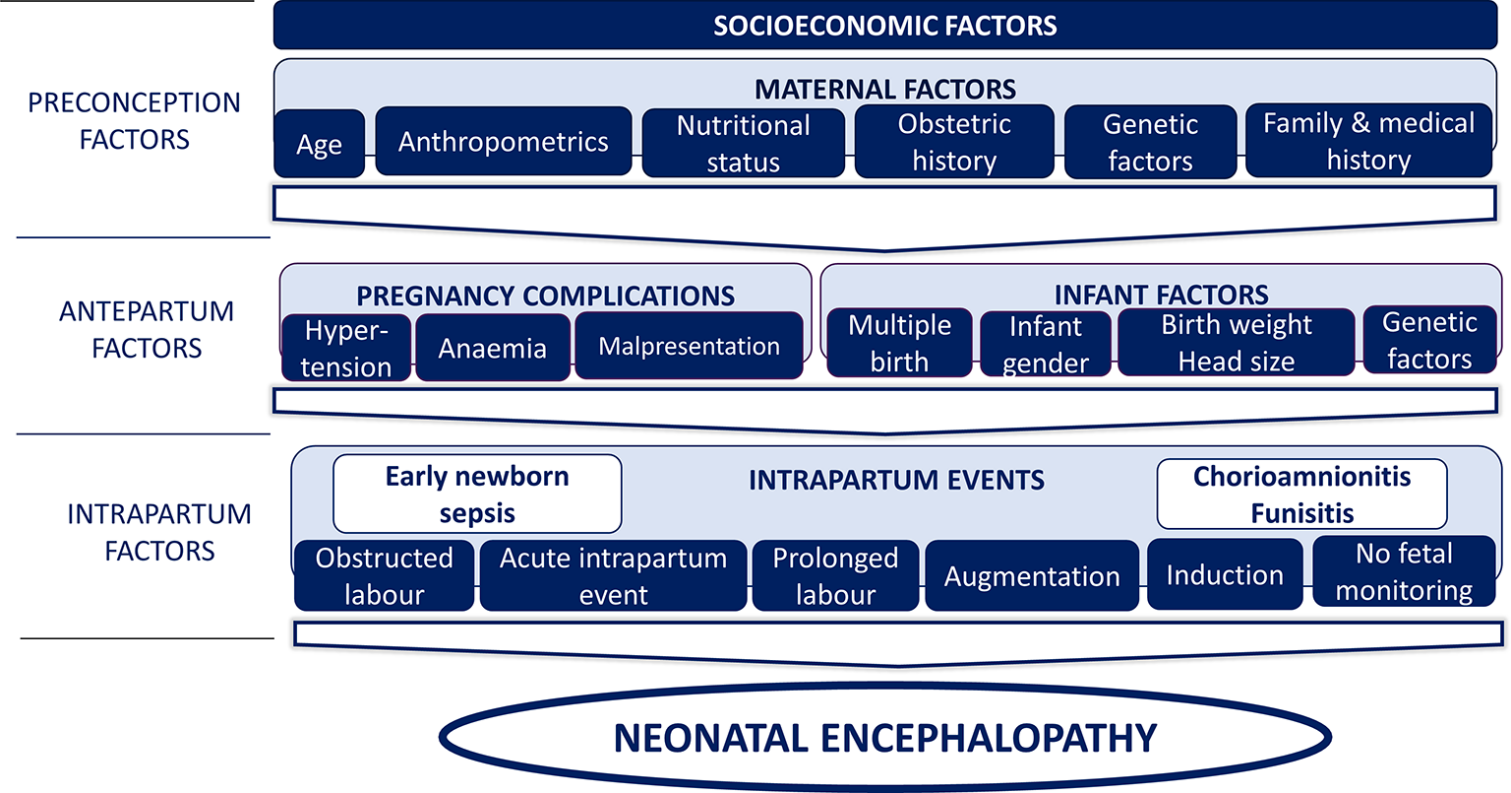
Conceptual framework of preconception, antepartum and intrapartum factors used for the multivariable modelling of risk factors for neonatal encephalopathy (NE). A three-phase, causal approach to multivariable modelling, based on a conceptual framework, was used to estimate adjusted ORs. First, a model was constructed to include preconception and antepartum variables associated with NE during univariable analysis (p<0.05) or where there was strong a priori evidence or biological plausibility for an association. Second, intrapartum variables were added to the model to examine the effect on the associations seen between the antepartum and preconception factors and NE and to examine the effect of adjusting for these factors on the associations between NE and intrapartum factors. Finally, associations between perinatal infection and inflammation exposures and NE were adjusted for peripartum factors found to be independently associated with NE in the second phase of the modelling. Factors considered to be a probable consequence of events related to NE, but not themselves causative of NE, that is, emergency caesarean section, meconium-stained liquor and poor Apgar scores, were not included in multivariable models.

All data were coded (CJT), double-entered (MS Access) and analysed using Stata V.11.0. Univariable logistic regression was used to calculate crude ORs and 95% CIs to identify the independent effects of variables. A three-phase, causal approach to multivariable modelling was used to estimate adjusted ORs (aORs) using the conceptual framework described in figure 2.

Since the proportion of missing data for variables was small (<5% for all variables, <1% for most variables), extra categories were created to represent missing values for the variables included. Inflammatory factors were defined as maternal CRP >90th centile, histological chorioamnionitis, histological funisitis, positive neonatal bacteraemia and raised neonatal CRP >97th centile. Population attributable fraction was calculated to assess the contribution of inflammatory factors to NE. The sample size of 210 cases and 409 controls ensured at least 80% power to detect risk factors conferring OR ≥2.5 at a significance level p<0.05, for main exposures with a prevalence of 5%–80%.

## Results

**Figure 3:**
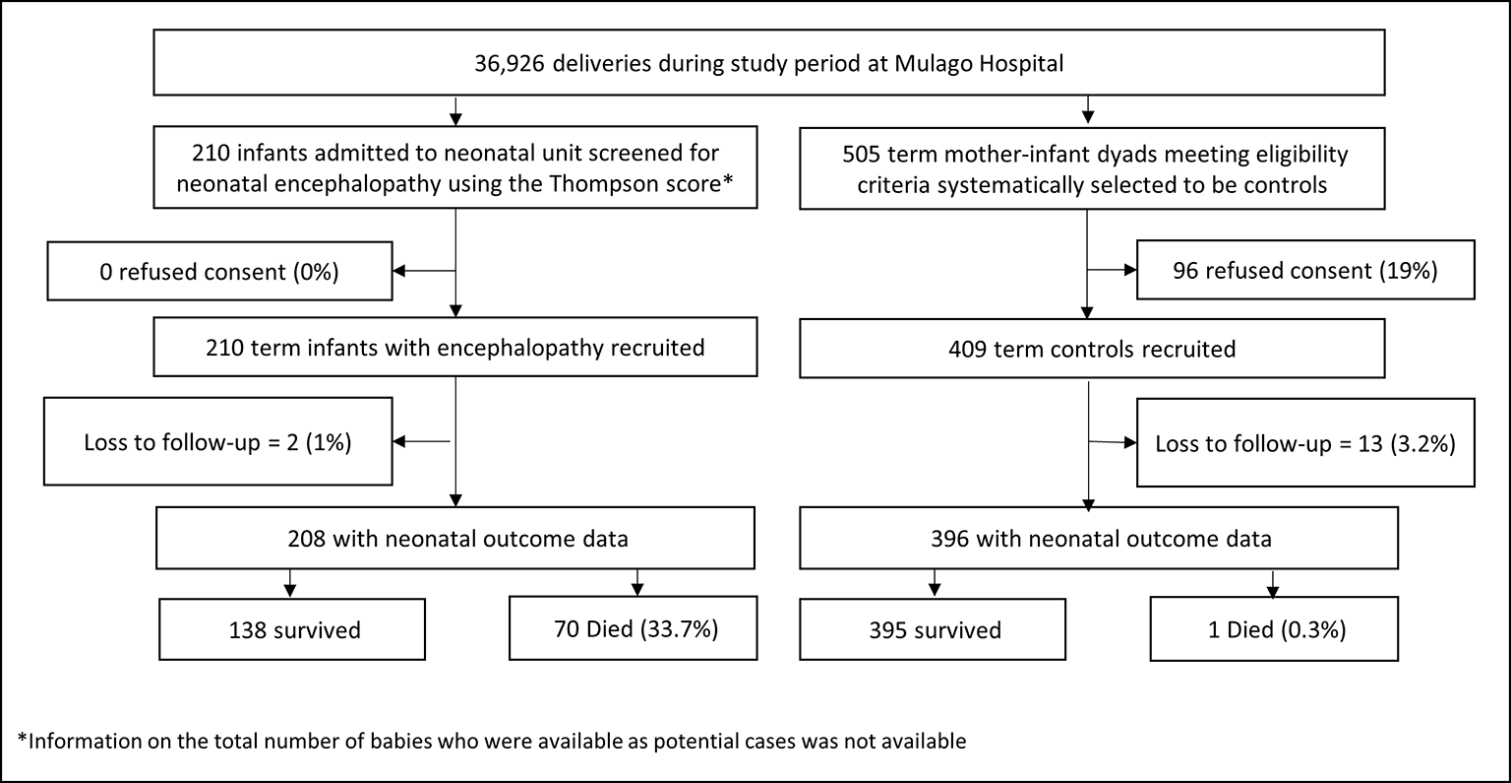
Flow diagram of participants.

During our 13-month recruitment period, 36 926 infants were born at Mulago Hospital, from which 210 encephalopathic and 409 control infants were recruited. Information on the number of babies who were considered as potential cases was not available due to the high number of babies admitted to the SCBU. For controls, a total of 505 mothers of term infants were identified by systemic sampling and approached. Of these 81% (409) consented to recruitment (figure 3).

Table 1 shows the early clinical characteristics of case and control infants. External signs of a major congenital abnormality were uncommon but more frequent in cases (2.9% (6/210) vs 0.7% (3/408), respectively, p=0.037). Losses to follow-up at 4 weeks were 1.0% (2/210) of cases vs 3.2% (13/409) of controls (p=0.103). Neonatal case fatality (<28 days) was 33.7% (n=70, 95% CI 27.2% to 40.1%) among encephalopathic infants (table 1).

**Table 1:** Early clinical characteristics of case and control infants

Clinical characteristics	Cases n=210 n (%)	Controls n=409 n (%)	p Value
Apgar 1 min			
≤3	68/202 (33.7)	2/405 (0.5)	<0.0001†
4–6	116/202 (57.4)	15/405 (3.7)	
≥7	18/202 (8.9)	388/405 (95.8)	
Apgar at 5 min			
≤3	8/185 (4.3)	1/399 (0.25)	<0.0001†
4–6	126/185 (68.1)	1/399 (0.25)	
≥7	51/185 (27.6)	397/399 (99.5)	
Need for any resuscitation			
No	6/162 (3.7)	331/378 (85.1)	<0.001
Yes	156/162 (96.3)	57/378 (15.1)	
Clinical features			
Grade of encephalopathy*			
Mild	25/210 (11.9)	–	
Moderate	114/210 (54.3)	–	
Severe	71/210 (33.8)	–	
Absent suck	166/209 (79.4)	–	
Clinical seizures	104/210 (49.5)	–	
Comatose	53/210 (25.2)	–	
Neonatal case fatality	70/208 (33.7)	1/396 (0.3)	<0.001

*Encephalopathy graded according to Sarnat & Sarnat.

†chi-squared for trendchi-squared for trend.

### Preconception, antepartum and intrapartum risk factors

In univariable analyses (table 2) case mothers were more likely to be primiparous, young, underweight and of short stature, to have severe anaemia or hypertension during pregnancy, and to be HIV-negative. Cases were more likely to be male, have a head circumference >97th centile and non-cephalic presentation. A third of encephalopathic infants were obstructed in labour compared with 8% of controls. Augmentation of labour, prolonged rupture of membranes, prolonged labour and meconium-stained liquor were all significantly more prevalent among cases. Acute intrapartum events were uncommon, but more prevalent among cases. No case infant was delivered by elective caesarean section. Fetal heart rate monitoring was significantly more prevalent among controls. In multivariable analysis one antepartum factor (hypertension in pregnancy), two infant factors (male sex, non-cephalic presentation) and four intrapartum factors (augmentation of labour, no fetal monitoring, acute intrapartum event and obstructed labour) were identified as independent risk factors for NE (table 2).

**Table 2:** Risk factors associated with neonatal encephalopathy in univariable analysis and after adjustment for preconception, antepartum and intrapartum factors

Risk factor	Cases n (%)	Controls n (%)	Unadjusted OR (n=620)	95% CI	Adjusted OR^‡‡^ (n=614)	95% CI
Sociodemographic factors						
Socioeconomic group						
High	33/209 (15.8)	82/405 (20.3)	1.00	–	1.00	–
Medium	131/209 (62.7)	245/405 (60.5)	1.64	0.87 to 3.11	1.52	0.81 to 2.34
Low	45/209 (21.5)	78/405 (19.3)	1.46	0.83 to 2.47	1.39	0.64 to 3.01
Preconception factors						
Maternal age <20 years	61/210 (29.1)	76/408 (18.6)	1.79	1.21 to 2.64	1.28	0.70 to 2.50
Maternal weight <50 kg	31/208 (14.9)	34/401 (8.5)	1.89	1.13 to 3.18	0.69	0.32 to 1.61
Maternal height <150 cm	40/208 (19.2)	44/399 (11.0)	1.92	1.21 to 3.06	1.48	0.73 to 3.00
Antepartum factors						
Primiparity	123/210 (58.6)	183/409 (44.7)	1.75	1.25 to 2.44	1.56	0.86 to 2.84
≥4 Antenatal visits	99/210 (47.1)	225/408 (55.2)	0.73	0.52 to 1.01	0.96	0.60 to 1.52
Previous ‘birth asphyxia’	20/87 (23.0)	33/226 (14.6)	1.75	0.94 to 3.25	1.27	0.50 to 3.21
Previous perinatal death	24/87 (27.6)	44/226 (19.5)	1.58	0.89 to 2.80	1.22	0.52 to 2.91
Severe anaemia during pregnancy*	10/209 (4.8)	5/408 (1.2)	4.05	1.37 to 12.01	4.38	0.97 to 19.82
Hypertension during pregnancy^†^	18/210 (8.6)	17/408 (4.2)	2.16	1.09 to 4.28	3.77	1.49 to 9.55
Maternal HIV-positive	15/210 (7.1)	53/409 (13.0)	0.52	0.28 to 0.94	0.57	0.24 to 1.32
Infant factors						
Male	136/210 (64.8)	196/409 (47.9)	2.00	1.42 to 2.82	2.51	1.55 to 4.07
Birth weight <2.5 kg	11/209 (5.3)	27/409 (6.6)	0.81	0.39 to 1.67	0.46	0.15 to 1.44
Birth weight >4.0 kg	12/209 (5.7)	12/409 (2.9)	2.00	0.88 to 4.51	2.17	0.77 to 6.13
Large head circumference‡	21/191 (11.0)	12/400 (3.0)	3.99	1.92 to 8.30	2.28	0.59 to 6.22
Twins	5/210 (2.4)	13/409 (3.2)	0.74	0.26 to 2.11	0.58	0.13 to 2.61
Non-cephalic presentation	23/209 (11.0)	11/409 (2.7)	4.47	2.14 to 9.37	5.74	2.01 to 16.41
Intrapartum factors						
Augmentation of labour	42/209 (20.1)	43/408 (10·5)	2.13	1.34 to 3.39	2.23	1.17 to 4.23
No auscultation of fetal heart rate during labour	114/208 (54.8)	115/408 (28·2)	3.09	2.18 to 4.38	2.75	1.71 to 4.22
Prolonged rupture of membranes^§^	21/193 (10.9)	15/396 (3·8)	3.10	1.56 to 6.16	2.44	0.90 to 6.60
Acute intrapartum event^¶^	8/210 (3.8)	4/409 (1·0)	4.01	1.19 to 13.47	8.74	1.70 to 45.02
Obstructed labour^**^	65/181 (31.9)	33/407 (8·1)	6.35	4.00 to 10.14	3.80	1.96 to 7.36
Prolonged labour^††^	100/190 (52.6)	157/387 (40·6)	1.63	1.15 to 2.31	0.81	0.47 to 1.39
Meconium-stained liquor	55/121 (45.5)	19/359 (5·3)	14.91	8.31 to 26.75	#	–
Elective caesarean section	0/209 (0.0)	3/409 (0·7)	–	–	#	–
Emergency caesarean section	50/209 (23.9)	56/409 (13·7)	1.98	1.30 to 3.03	#	–

*Defined as haemoglobin ≤7 g/dL during pregnancy and documented in the clinical record.

†Defined as systolic blood pressure >140 mm Hg or diastolic >90 mm Hg developing after 20 weeks’ gestation.

‡Defined as >97th centile in the control population (37.9 cm).

§Defined as rupture ≥24 hours.

¶Defined as antepartum haemorrhage, cord prolapse or uterine rupture.

* *Defined as labour with no advance of the presenting part despite strong, regular uterine contractions as documented in the intrapartum record.

††Defined as >24 hours in primiparity and >12 hours in multiparity.

‡‡Adjusted for all other variables in the table plus maternal C reactive protein >90th centile and neonatal bacteraemia. Factors considered a consequence of intrapartum events (#) were not included in the model.

### Perinatal infection/inflammation risk factors

The presence of neonatal bacteraemia was examined in 210 cases and 105 controls. For controls, quantitative polymerase chain reaction (qPCR) was performed among the first 101 recruits. In a further 4, qPCR and blood cultures were performed due to clinical concerns of early possible severe bacterial infection; all were negative. No significant differences were seen between control infants with and without bacteraemia results with respect to demographic or other baseline characteristics (data not shown). The prevalence of pathogenic bacterial species among infants with NE was 3.6%, 6.9% and 8.9%, with culture, PCR and both tests in combination, respectively.^20^ More encephalopathic infants than controls had pathogenic bacterial species detected (8.9% vs 2.0%, p=0.028) using culture and PCR in combination.^20^ PCR detected bacteraemia in 11 culture-negative encephalopathic infants (3 group B Streptococcus, 1 group A Streptococcus, 1 *S. aureus* and 6 Enterobacteriaceae (2 *Enterobacter sp,* 1 *Pantoea sp,* 1 *Escherichia coli* and 2 identified only as Enterobacteriaceae species)).^20^ Three case infants were negative on PCR but blood culture was positive for *S*. *aureus*. Coagulase-negative staphylococcus was considered not pathogenic.

Placentas were processed and reported in 28.6% (60/210) of cases and 24.9% (102/409) of controls. No significant differences were seen between mothers with and without placental samples with respect to demographic or other baseline characteristics (data not shown). After adjustment for preconception, antepartum and intrapartum risk factors (detailed in table 3), several markers of infection or inflammation were found to be highly significant risk factors for NE, including neonatal bacteraemia, histological funisitis and raised maternal and early neonatal CRP (table 3).

**Table 3:** Univariable and adjusted analyses of perinatal infection/inflammation risk factors and between inflammatory and probable asphyxial factors

			Univariable analysis		Multivariable analysis	
Risk factor	Case n (%)	Control n (%)	Unadjusted OR	95% CI	Adjusted OR^†^	95% CI
Maternal and newborn C reactive protein (CRP)						
Maternal CRP						
<10th centile (≤4.7 mg/L)	3/205 (1.5)	40/407 (9.8)	1.00	–	1.00	–
10th–90th centile (4.7–86.6 mg/L)	131/205 (63.9)	327/407 (80.3)	5.34	1.62 to 17.57	5.21	1.23 to 22.06
>90th centile (>86.6 mg/L)	71/205 (34.6)	40/407 (9.8)	23.67	6.87 to 81.42	37.16	7.86 to 175.66
Neonatal CRP						
<90th centile (<6.6 mg/L)	159/205 (77.6)	359/398 (90.2)	1.00	–	1.00	–
90th–97th centile (6.6–31.7 mg/L)	29/205 (14.2)	28/398 (7.0)	2.34	1.35 to 4.06	3.17	1.57 to 6.41
>97th centile (>31.7 mg/L)	17/205 (8.3)	11/398 (2.8)	3.49	1.60 to 7.62	9.52	3.33 to 27.21
Maternal and newborn infection and inflammation						
Neonatal bacteraemia*	18/210 (8.6)	2/105 (1.9)	4.83	1.10 to 21.22	8.67	1.51 to 49.74
Histological chorioamnionitis alone (no funisitis)	10/60 (16.7)	11/102 (10.8)	2.33	0.91 to 5.98	3.20	0.66 to 15.52
Histological funisitis (with chorioamnionitis)	16/60 (26.7)	4/102 (3.9)	10.24	3.19 to 32.82	11.80	2.19 to 63.45

*Defined as blood culture and/or species-specific bacterial quantitative Polymerase Chain Reaction (qPCR) for an organism known to be pathogenic among term newborns.

†Adjusted for preconception/antepartum factors in table 2, plus intrapartum factors (augmentation of labour, prolonged labour, obstructed labour and acute intrapartum event).

In NE, neonatal case fatality was not significantly higher for infants with early bacteraemia versus those without (44.4% (8/18) with bacteraemia vs 32.6%% (62/190) in those without, OR 1.65 (0.62–4.39), p=0.32). Intrapartum antibiotic was commonly used in cases and controls (17.8% (37/210) vs 14.0% (57/408), respectively, p=0.213). Among encephalopathic infants, intrapartum antibiotic use was not associated with improved neonatal survival (case fatality with intrapartum antibiotics 32.4% (12/37) vs 34.3% (58/169) without, p=0.826).

## Discussion

Few studies have examined perinatal risk factors for NE in sub-Saharan Africa. In this Ugandan population, we found that maternal and newborn infection and inflammation, based on blood cultures, molecular assays and a subset of placentas, are independent risk factors for NE, with the strongest associations seen with fetal inflammation (funisitis) and early neonatal bacteraemia. Neonatal case fatality was not significantly higher for infants with NE exposed to neonatal bacteraemia. Intrapartum antibiotic use did not improve survival among encephalopathic infants. Other potentially modifiable antepartum and intrapartum risk factors were identified, with many antepartum factors mediated by intrapartum events suggesting potential large gains with improvements in intrapartum care.

In our study, funisitis, or infiltration of the umbilical cord with acute fetal inflammatory cells, was a significant risk factor for NE; however, the presence of membrane inflammation alone (isolated histological chorioamnionitis) was not. The reduced sample size of infants with placentas collected (n=162) may have been responsible for the lack of significance of the association seen between NE and chorioamnionitis; however, this smaller sample size retained more than 80% power to detect the associations seen for funisitis. Triggering of the fetal inflammatory responses, as evidenced by the presence of funisitis, has been associated previously with neurological impairment among both term and preterm infants.^22–25^ A high incidence of histological funisitis among infants with NE has been previously reported (22%–31%),^24 26^ with one retrospective observational study also finding funisitis to be significantly associated with encephalopathy (OR 9.29).^24^ A recent study from the Netherlands, examining associations between placental pathology and pattern of brain injury in NE, found a high incidence of histological chorioamnionitis across all patterns of brain injury, when compared with healthy term deliveries in a historic cohort from the same centre (50% vs 18%, respectively).^26^ The stronger associations with NE seen for histological funisitis, as opposed to chorioamnionitis, may imply that activation of the fetal inflammatory response is a key event, or that duration of exposure, or proximity of inflammation to the fetus, may be important.

Both a sensitising and preconditioning role of bacterial endotoxin on the effect of hypoxia-ischaemia on the immature brain have been seen in animal models, but supporting clinical data are limited. Our study provides evidence of an important role for early newborn bacteraemia in NE in humans, with an eightfold increase in odds of NE. Early neonatal infection did not significantly increase the odds of death among infants with NE, although the small numbers of infants in the infection-exposed group (n=18) may have reduced the ability to detect a true difference. Our findings are supported, however, by a US study, examining MRI in predicting outcome after NE, that reported a similar prevalence of sepsis and an increase in adverse neurodevelopmental outcomes, but not death, among encephalopathic infants with sepsis.^27^ Despite the administration of intrapartum antibiotics in almost a fifth of encephalopathy cases in our study, antibiotics did not improve survival. This lack of effect of antibiotic therapy is consistent with findings from the ORACLE trial, which examined neonatal outcomes after preterm rupture of membranes.^28 29^ Although our infants were not cooled, our incidence of sepsis was comparable to many hypothermia trials from high-income settings (5%–14%).^30^

Other studies from both low-income and high-income settings have examined this ‘dual hit’ hypothesis of infection and hypoxia-ischaemia. A population-based study from South Asia also reported significantly increased odds of early neonatal death due to intrapartum asphyxia in newborns exposed to prolonged rupture of membranes (aOR 1.52; 1.15–2.02),^31^ a predisposing factor for intrauterine infection and early newborn bacteraemia. Previous studies in high-income settings have shown a high prevalence of inflammatory indicators among infants with NE,^32^ and a combination of antenatal infection and a potentially birth-asphyxiating condition can dramatically increase the risk of cerebral palsy (OR 78.0) when compared with either alone.^33^ In our study, positive dose-related associations were seen between raised maternal and neonatal CRP, an acute phase reactant and NE. Previous studies have shown associations between a raised neonatal CRP and a number of neonatal condition including infection, perinatal asphyxia and meconium aspiration,^34^ and so an association with maternal and newborn infection cannot be assumed.

In univariable analysis, we identified several other peripartum risk factors for NE. Maternal and infant factors included primiparity, young maternal age, low maternal weight or stature, complications during pregnancy (hypertension, severe anaemia) and large infant head circumference. However, these antepartum factors did not significantly improve the fit of the data after adjustment for intrapartum events, suggesting that their association with NE is likely mediated by increasing the risk of intrapartum complication. However, even after adjustment, male infants and those of non-cephalic presentation remained significant risk factors for NE. Although an acute intrapartum event significantly increased the risk of NE, such events were uncommon, and other potentially modifiable factors such as hypertension in pregnancy, lack of fetal monitoring, non-cephalic presentation and obstructed labour contributed to a higher proportion of cases of NE in our population. A high demand for services in the face of low midwife to mother staffing ratios, lack of equipment and delays in providing rapid obstetric intervention may have been substantial contributing factors in our setting. Prioritising effective risk assessment and intrapartum care for women and babies delivering in healthcare facilities in low-resource settings has the potential to substantially reduce neonatal risk.^35^

There were limitations to this study. Placental sampling was performed in only a quarter of women, and although sampling appeared non-differential all bias cannot be excluded, and power to detect associations for placental variables was reduced. Recruitment of participants from a high-risk referral centre may explain the high rates of chorioamnionitis seen; however, this would have led to a conservative estimate of all ORs. Although we performed species-specific bacterial PCR in conjunction with neonatal blood culture to strengthen the diagnosis of neonatal bacteraemia, we still cannot exclude underdiagnosis. The majority of control infants did not show clinical signs of sepsis, and loss to follow-up rates in both groups were low. We cannot exclude all selection and recall bias, although rigorous verification with hospital records and comprehensive staff interview training aimed to minimise this.

In summary, perinatal infection and inflammation are independent risk factors for NE in this low-resource setting, supporting a role in the aetiological pathway of term brain injury. Intrapartum antibiotic administration did not mitigate again adverse outcomes. The importance of intrapartum risk factors in this sub-Saharan African setting is highlighted.
